# Designing their Own Story: A Meta-Ethnography of Health Promotion Among Adolescents with Parental Substance Use Problems

**DOI:** 10.1177/14550725261436976

**Published:** 2026-04-17

**Authors:** Signegun Romedal, Anne Schanche Selbekk, Siri Håvås Haugland, John-Kåre Vederhuus, Bente Birkeland

**Affiliations:** 1 Department of Psychosocial Health, University of Agder, Grimstad, Norway; 2 Sørlandet Hospital, BarnsBeste - National Competence Network for children as next of kin, Kristiansand, Norway; 3 Department of Public Health, University of Stavanger, Stavanger, Norway; 4 Center for alcohol and drug research (KORFOR), Stavanger University hospital; 5 Sørlandet Hospital, Addiction Unit, Kristiansand, Norway

**Keywords:** adolescents, health promotion, parental, salutogenesis, substance use problem

## Abstract

**Aims:**

Children and adolescents living with parental substance use problems (PSUP) are at potential risk of developing cognitive, behavioral, psychosocial and emotional difficulties affecting their health. However, little is known about how they themselves understand and promote their own health and well-being. This meta-ethnography aims to integrate and synthesize qualitative studies that have explored – from a salutogenic health-promoting perspective – how adolescents living with PSUP express health promotion and well-being, focusing on resources for positive health.

**Methods:**

The seven iterative phases by Noblit and Hare were followed. Systematic literature searches yielded 13 articles.

**Results:**

**“**Designing their own story” was identified as an overarching metaphor accentuated by three main themes that we named: (1) “controlling before opening up”; (2) “choosing trusted support”; and (3) “learning for life”. The overarching metaphor symbolizes how adolescents living with PSUP demonstrate agency in a stressful life situation, expressing a sense of control in everyday life and actively choosing whom to involve for support.

**Conclusions:**

Supplementing protection and prevention, a different view on narrative of adolescents living with PSUP emerges in the conceptual framework of salutary health promotion. When support is offered, this can inform professionals, service providers and policymakers about the significance of acknowledging and exploring the resources within the adolescents themselves and in their environments. Further research is needed to gain a broader and deeper understanding of how to support adolescents living with PSUP in a health-promoting direction, recognizing their potential to improve their health and well-being in their stressful and challenging life situation.

## Introduction

Substance use problems represent a significant public health challenge with far-reaching impacts that extend to the children of those affected (European Monitoring Centre for Drugs and Drug Addiction, 2010; World Health Organization (WHO), 2019, 2024). Children (aged 0–18 years) living with parental substance use problems (PSUP) are at potential risk of developing cognitive, behavioral, psychosocial and emotional difficulties(Adamson & Templeton, 2012;Omkarappa & Rentala, 2019;Solis et al., 2012; Velleman & Templeton, 2016) and are at risk of intergenerational transmission of substance use problems (Haugland et al., 2015; Latvala et al., 2022; Straussner & Fewell, 2018).

In this study, PSUP refers to parents’ display of substance use disorder, including alcohol, as well as harmful and problematic use of substances (American Psychiatric Association, 2022; Birkeland and Weimand, 2015; Laslett et al., 2011; Hope 2014). PSUP also refers to situations where children perceive the substance use as problematic regardless of whether or not the parent has a diagnosis (Hansen, 1994; Lindgaard, 2008). Children in such situations can struggle to function in what they considered a normal family everyday life (Bönnhoff & Larsen, 2014; Lindeman et al., 2022; Tamutienė & Jogaitė, 2019; Werner & Malterud, 2016), while trying to manage and mitigate vulnerabilities and be resilient to unpredictable, adverse and often stigmatizing experiences (Muir et al., 2023; Park & Schepp, 2015). They are shown to frequently experience feelings of shame and guilt; to disguise their parents’ problems (Ytterhus et al., 2024) and they are also more likely to be subjected to violence and abuse (Backett-Milburn et al., 2008; Hopkins et al., 2024).

It is well documented that experiences of PSUP have negative consequences for health during both childhood and adulthood (Lund et al., 2020; Haugland et al., 2020; Muir et al., 2023; Raitasalo & Holmila, 2017). Resilience is a resource-oriented concept related to PSUP, which concerns how children and young people show few or no symptoms despite risk exposure – often through problem-solving skills and protective internal and external factors (Ahlborg et al., 2024; Park & Schepp, 2015). Protective factors can be individual or found outside the immediate family; they include self-efficacy, self-disclosure, coping strategies, personal competence and support from extended family members or teachers (Ahlborg et al., 2024; Park & Schepp, 2015; Velleman & Templeton, 2016; Wlodarczyk et al., 2017).

Although many studies have shown the protective impact of resilience and that PSUP has a negative health-related impact, health is in itself a multifaceted concept. It is challenging to provide precise definitions of health, as it is perceived subjectively by the individuals and interpreted differently across individuals and contexts (Cross & Woodall, 2024). This study is grounded in the premise that health promotion – as defined in the WHO's Ottawa Charter for Health Promotion – is “the process of enabling people to increase control over, and to improve, their health” where health is recognized as subjective, a resource for everyday life and a human right emphasizing the interaction between individuals and their environment (WHO, 1986).

In health promotion, salutogenesis may offer a relevant perspective (Antonovsky, 1996; Eriksson & Lindström, 2010, 2014; Vinje et al., 2022). A salutogenic perspective sees stressors as universal and inevitable and, for example, ubiquitous microbiological and sociocultural stressors as a normal part of life. Chaos and change (heterostasis) and experiences of illness or suffering (entropy) are inherent and entangled aspects of human existence. The fundamental contribution of salutogenesis to health promotion has been to raise the philosophical question of what creates health and explore “the origin of health”, complementing pathogenesis which is focused on causes of disease, distress and risk factors (Antonovsky, 1987; Mittelmark & Bauer, 2022).

Five aspects characterize a salutogenic orientation (Antonovsky, 1987; Vinje et al., 2022). First, health is viewed as a position along a health ease/dis-ease continuum where individuals constantly move between maximal illness and maximal wellness, with a focus on the direction toward the “ease” end. A second aspect is the holistic view of individuals and their life stories. The third aspect highlights health-promoting factors, the resistance resources. The fourth aspect is that stressors and tension may be pathogenic, neutral or health-promoting, contingent upon the availability and use of internal and external resources, as well as the individual's process of meaning making. The fifth aspect relates to the individual's capacity to actively adapt to changing circumstances and challenges. Salutogenesis is concerned not only with health and the movement toward health, but also with the enhancement of well-being. From a salutogenic perspective, health is understood as a process and capacity to activate resources for coping, and well-being concerns experience and quality, a subjective experience of coping and having a good and meaningful life (Vinje et al., 2022).

The above illustrated complex health ontological perspective serves as a backdrop for our study exploring adolescents’ health promotion in the context of PSUP. Adolescence is the transitional phase between childhood and adulthood which lays the foundation for lifelong health. It is a pivotal stage in which physical, cognitive and psychosocial development profoundly shapes how individuals feel, think, make decisions and engage with their environment. Adolescents’ evolving capacities shape their perception of health and future projects, as well as the factors influencing their decisions and behaviors. All of this has implications for the types of interventions that are needed and implemented (WHO, 2014). Exploring health promotion for adolescents living with PSUP from their own perspective is therefore relevant to enhance the current understanding of how to support them to move in a health-promoting direction given their life situation.

Municipalities in Norway are responsible for promoting health among children and young people (Helse- og omsorgstjenesteloven (Health and Care Services Act), 2011,§ 3-3), and the responsibility of healthcare professionals is to help ensure that children of parents with health issues receive care and follow-up (Helsepersonelloven [Health Personnel Act] 1999, § 10a). However, knowledge in this area is scarce, with few qualitative meta-syntheses focusing on the experiences and needs of children living with PSUP (Ahlborg et al., 2024; Muir et al., 2023; Ruud et al., 2015; Selbekk et al., 2019). Although resilience has been described in research, to our knowledge, no meta-synthesis has specifically focused on the salutogenic health-promoting perspective highlighting resources for adolescents living with PSUP.

The aim of this meta-ethnography is therefore to expand the existing knowledge through integrating and synthesizing qualitative studies on adolescents’ experiences living with PSUP from a salutogenic health-promoting perspective, focusing on resources for positive health. Thus, the review question is: How do adolescents living with PSUP express their health promotion and well-being?

## Methods

To explore and articulate adolescents’ expressions of their health promotion and well-being, we used meta-ethnography (Noblit & Hare, 1988). Meta-ethnography is a well-established interpretive meta-synthesis approach for qualitative evidence synthesis in health science, using qualitative studies on a phenomenon as data (Bondas, 2025; Lindeman et al., 2022). This methodological choice is grounded in our recognition of the scarce research available concerning adolescents living with PSUP from a health promotion perspective. What is unique about meta-ethnography is the translation theory of social explanation based on a comparative, interpretative understanding rather than aggregation (Noblit & Hare, 1988), with results greater than the sum of its parts, as each phase in the interpretive process involves an increased integration of data (Bondas, 2025). An essential element is to determine how findings from the included primary studies relate to one another, as they have been interpreted and presented by their authors using metaphors, referring to key terms and concepts communicating meaning (Noblit & Hare, 1988). The translation (the analyses) and synthesis create a dynamic, non-linear and iterative research process of interpreting, reflecting and theorizing. In line with France et al. (2019), this method can elicit novel interpretations and generate new overarching knowledge to inform and improve practice, services and policy by synthesizing the logics described in the scientifically reviewed studies, lifting the described meaning-making to a more principle level of understanding using metaphors. For this study, the meta-ethnography was conducted through an iteratively driven process consisting of seven non-linear and overlapping phases (France et al. 2019; Noblit & Hare, 1988). A visual presentation of the phases in meta-ethnography is provided in the Supplementary material (File S1).

### Searches and Critical Appraisal

#### Getting Started

A research team was established to support a critical and reflective research process (France et al., 2019); the members consisted of persons active in five distinct professions with theoretical and clinical backgrounds in health, social work and sociology in domains concerned with substance use and children as next of kin. The research team had diverse methodological expertise complemented by a wide array of personal experiences.

#### Deciding What is Relevant

In the next phase, inclusion and exclusion criteria were established in alignment with the review question through consultations among three of the reviewers (S.R., B.B.j and A.S.S.); these criteria were further elaborated during the screening process. As this meta-ethnography is focused specifically on knowledge concerning PSUP, studies concerning other parental conditions and comorbidities were excluded. The inclusion criteria used in the study selection are presented in Table 1.

**Table 1.** The Inclusion/Exclusion Criteria.

Inclusion	Exclusion
Adolescents (aged 10–19 years) living with PSUP (Alcohol, illegal drugs, and addictive medications) Young people (aged 20–25 years) reflecting retrospectively on their adolescence Studies addressing perspectives of adolescents and young people Qualitative studies and qualitative data from mix-methods studies Peer-reviewed original qualitative research studies No geographical restriction No publication date restrictions Studies published in English	Parents’ or professionals’ experiences Studies focusing exclusively on risks or disease prevention related to adolescents living with PSUP Studies with parental co-morbidity to other disorders or illnesses Quantitative studies Grey literature, theoretical papers, reviews, editorials, book chapters, or comments Studies published in languages other than English

PSUP = parental substance use problems.

A systematic literature search strategy was devised with support from a medical librarian. After several test searches which yielded no relevant results concerning health promotion and salutogenesis in relation to adolescents living with PSUP, we finally searched for the following: keywords and controlled vocabulary (MeSH terms, CINAHL headings, PsycInfo psychological Index Terms) entered individually, in combinations, with full spellings and with truncation: child; offspring; adolescent; teenager; youth; young; son; daughter; alcoholic; parent; maternal; paternal; mother; father; drinking; use; abuse; misuse; dependent; or addiction; substance; drug; sedative; opiate; and opioid. Restrictions were placed to locate only English, peer-reviewed, qualitative, and interview-based studies. We systematically searched for qualitative studies in CINAHL, Embase, PsycINFO, Academic Search and Web of Science. No restrictions regarding publication year or geographical location were applied, thus ensuring a comprehensive overview of the existing evidence base. The search was conducted during the period 29 February 2024 to 18 April 2024 (for the complete search strategy, see the Supplementary material, File S2).

**Figure 1.**
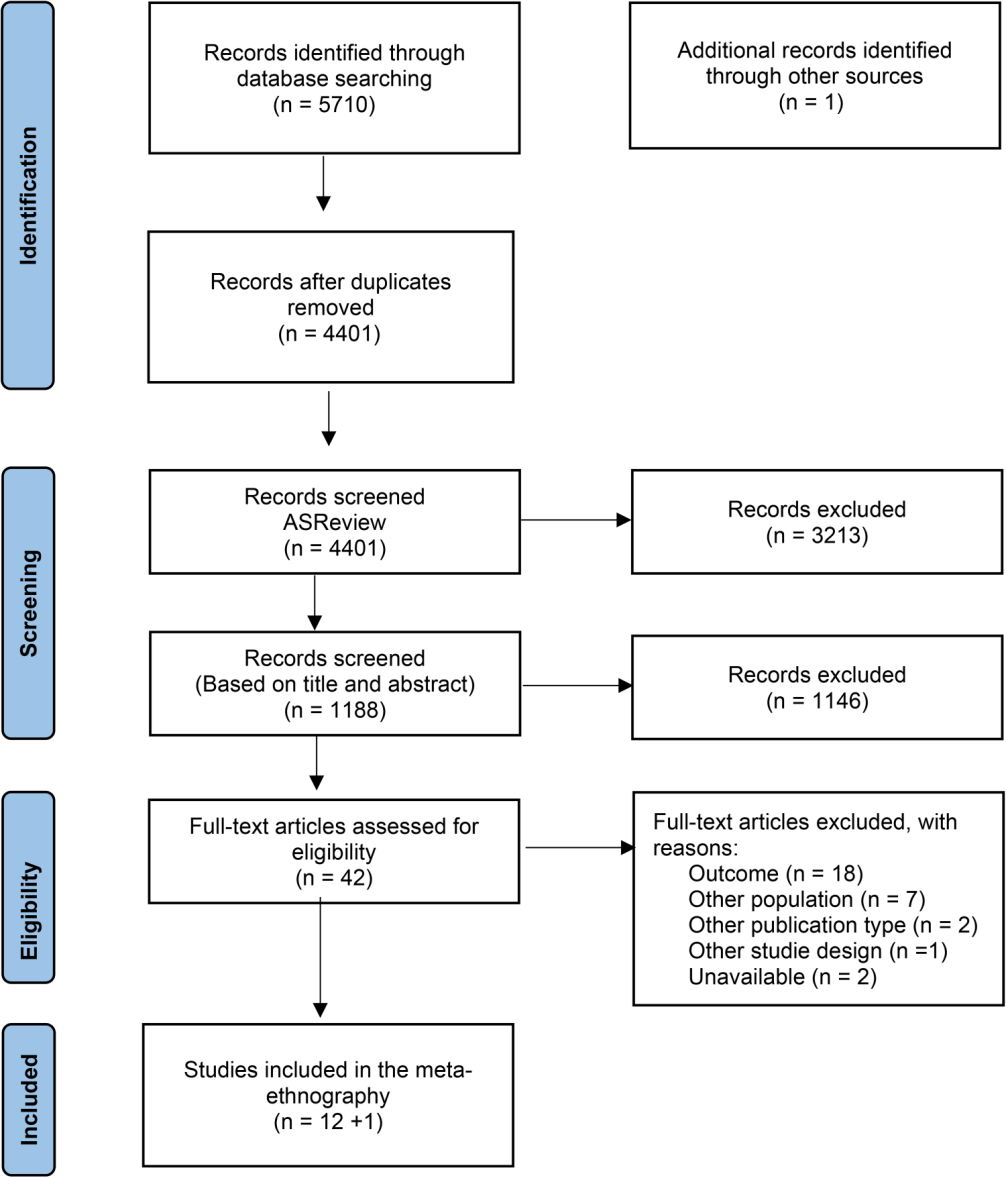
An adapted PRISMA flow-chart of the literature (Page et al., 2021).

The systematic search resulted in 5710 records including 1309 duplicates. In the initial screening phase, we used the AI tool ASReview (van de Schoot et al., 2021) to exclude studies that clearly represented irrelevant content, such as pregnancy. We uploaded 4401 papers starting to mark a few as relevant or irrelevant. Subsequently, ASReview suggested the next most likely relevant papers based on our previous labels, which we thoroughly assessed, resulting in a total of 1188 papers getting screened. Second, S.R. and B.B. blindly reviewed the 1188 titles and abstracts before deciding whether to include or exclude the papers screened, focusing on resources using the reference management tool Rayyan. Based on an initial screening of titles and abstracts after removing some more duplicates, we identified 42 articles for further review. Then, S.R., B.B., and A.S.S. blindly reviewed the 42 qualitative studies, out of which 12 were included. In addition, one hand-searched reference was included. Minor discussions related to study design, study quality and perspective of health represented through the selection process were resolved through re-reading and deliberations within the research team. The outcome of study selection (Figure 1) is reported in line with the PRISMA flowchart (Page et al., 2021).

Furthermore, S.R., B.B., and A.S.S. read the full texts of the 13 articles for quality appraisal in line with CASP Qualitative Studies Checklist (CASP, 2018), finding all studies to be relevant and trustworthy (see Supplementary material, File S3). Articles that were hard to determine were re-read by at least two of the reviewers. An overview of the final 13 studies and their characteristics is presented by numbers in Table 2.

**Table 2.** Characteristics of Included Studies.

First author and title	Country	Design	Aim/objective	Sample	Results
**1.** **Alexanderson & Näsman (2017)** Children’s experiences of the role of the other parent when one parent has addiction problems	Sweden	Semi- structured interviews	To describe and problematize the role of the other parent in relation to the children when one parent has addiction problems, from a child perspective	*n* = 23 10 aged 6–12 years 13 aged 13–19 years	The other parent is a source of support, help and protection to some children, but difficulties may reduce the other parent's ability to protect the children. The other parent is an important adult in their formal social network. The young people (compared to children) mostly describe the other parent as supportive
**2.** **Bickelhaupt et al. (2021)** The Influence of Parental Alcoholism on Parent-Adolescent Relationships From Adolescence Into Emerging Adulthood: A Qualitative Inquiry	USA	In-depth interviews	To provide a novel, in-depth perspective to some of the positive and adaptive developmental strategies used by emerging ACOAs perspective which contribute to successful functioning as an emerging adult in terms of internalizing and externalizing behaviors	*n* = 13 aged 21–25 years Girls 9 Boys 4	Four key themes were presented : (1) Family of Alcoholism, (2) Exhibited Adolescent Behaviors (3) Resilience Path to Functional Development (4) Current Drinking Parent Relationship. Individuals gained better control of their environment by emotionally and physically detaching from a parent's alcoholism and accepting that the behavior was not their responsibility
**3.** **Hagström & Forinder (2022)** If I whistled in her ear she’d wake up’: children’s narration about their experiences of growing up in alcoholic families	Sweden	Narratively structured interviews	To investigate what it means to grow up in an alcoholic family environment	*n* = 19 aged 16–24 years at the third interview Girls 8 Boys 11	1. The children positioned themselves as “vulnerable victims” exposed to their parents’ alcoholism and to situations of severe neglect, domestic violence and sexual abuse. They experienced powerlessness and lack of resources for coping with emotional distress and risk, and an urgent need for protection and care. 2. The children positioned themselves as “competent agents” who had developed purposeful strategies for managing their life situation, trying to reduce their parents’ drinking and took the role as a “young carer”. They tried to normalize themselves in their social circle as “silenced and invisible victims”
**4.** **Holmila et al. (2011)** Invisible victims or competent agents: Opinions and ways of coping among children aged 12-18 years with problem drinking parents	Finland	Web-based questionare	To describe the lives of children with problem drinking parents from children's own perspective, emphasizing their experiences, agency and coping	*n* = 70 aged 12–18 years Girls 58 Boys 12	Children with problem drinking parents are a hidden population and neglected by services. Children have developed their own ways of trying to cope. They have various practical suggestions and opinions concerning services and useful types of help
**5.** **Johnson (2013)** African American Adolescents’ Interactions With Their Substance-Using Mothers	USA	In-depth, semi- standardized interviews	To explore youth reports of their interactions with their substance-using mothers to determine whether the interactions (a) shape youths’ perceptions of maternal influence, (b) reveal patterns of communication, (d) lead youth to assume specific roles within the family and (d) affect youths’ involvement with other social support networks	*n* = 15 aged 20–25 years Female 9 Male 6	Four themes were identified: (1) Influence, (2) Communication, (3) Assumed roles and (4) Support networks. Youth report maternal influence on their attitudes in general and toward child-rearing and peer group affiliations. Communication between the dyads is often strained and youth assume roles of sibling protector and maternal confidant. Youths have supportive adults in their lives but their engagement with this support is also shaped by maternal substance use
**6.** **Mushonga & van Breda (2021)** Nonhuman systems as a source of interactional resilience among university students raised by alcohol-abusing caregivers in Lesotho	Lesotho	Semi-structured interviews and arts-informed “draw-and write"	To explore the interactions of resilient adult children of alcohol abusing caregivers with non-human systems to understand what nonhuman systems they engage with and how these interactions operate to promote resilient outcomes	*n* = 15 aged 20–25 years Female 9 Male 6	Two themes were presented: (1) Interacting with empowering messages from non-present writers (through songs and books) and inspirational speakers (through videos) and (2) interacting with imaginary friends and inanimate objects (dolls and tattoos) to enhance their resilience
**7.** **Mushonga & van Breda (2023)** Other-initiated interactions that contribute to resilient outcomes among young adults raised by caregivers who misuse alcohol	Lesotho	Semi-structured interviews and arts-informed “draw-and write"	To explore the interactional resilience of adult children raised by caregivers who misused alcohol, specifically focusing on the interactions of these young adults with people in their environment who initiated support that enriched their resilience and how these other-initiated interactions function to promote positive outcomes	*n* = 15 aged 20–25 years Female 9 Male 6	Three themes were formulated: (a) Other-initiated material support that elicits a response of resolve or obligation towards the other, (b) Other-initiated sustained emotional support that elicits a response of hope for the future, (c) Other-initiated challenging support that elicits a change in behavior. Interactional resilience results when people in a child's environment are attentive, responsive and willing to engage with the child and when the child is receptive to offers of support and able to engage with them meaningfully
**8.** O’Connor et al. (2014) Perspectives on children’s experiences in families with parental substance misuse and child protection interventions	UK	Mixed methods, In-depth interviews	To provide perspectives on the experiences of children and young people living in families with parental substance misuse, drawing on the views of young people who provide first person retrospective accounts of living with parental drug and alcohol use	*n* = 27 Families *n* = 5 Young people	Four key themes were presented : (1) Living with neglect, trauma and violence, (2) Living with tension, (3) Attachments and consistency, (4) Childhood & adult difficulties, (5) Protective role & sense of agency and (6) Aspiration, success and helpful interventions. The family environment was typified as extremely challenging. A proportion of parents identified reduced substance misuse and, in some cases, improved family functioning. Most families experienced emotional, social and psychological difficulties
**9.** **Offiong et al. (2020)** «I missed open arms»: The need for connectedness among Black youth affected by parental drug use	USA	In-depth interviews	To explore connectedness among black youth affected by parental drug use and identify the consequences of when connectedness is missed	*n* = 30 Parents 11 Youth aged 18–24 years 14 Youth providers 5	Three key themes presented: (1) Missing parental connections, (2) The desire for consistent, trusted adults and (3) The consequences of missed connections. All participants emphasized the limited emotional support and guidance provided to through affected by parental drug use. Extended family members and community mentors stepped in to fulfill unmet needs, when possible
**10.** **Park & Schepp (2017)** The Patterns of Adaption While Growing Up Under Parental Alcoholism: A Grounded Theory	South Korea	Face-to-face or telephone-based semi-structured interviews	To explore the psychosocial processes that Korean children, who grew up with a father with alcohol dependence, have undergone since they were very young and how they adapt to living their own lives	*n* = 20 Mean age 24.55 years (range 19-30 years), 13 Female 7 Male	The participants showed a similar pattern of feelings, emotions and coping behaviors while growing up, describing this process as “the adaptation process of separating their own identity from their fathers” in six stages: being trapped, awakening, struggling, blocking, understanding and separating.
**11.** **Templeton et al. (2011)** Young people’s views on services to help them deal with parental substance misuse	UK	Face-to-face interviews	To present young people’s views on three new family-focused services	*n* = 23 aged 10–17 years Female 11 Male 12	Six themes presented: (1) Receiving a service, (2) Relationships, (3) Facilitating change, (4) Change in personal values and (5) Change in family life and the future. Young people benefitted through meeting other people and having an opportunity to talk and share experiences, learning about addiction and understanding and controlling their emotions. Their families became safer, healthier and more cohesive
**12.** **Tinnfält et al. (2011)** Adolescent Children of Alcoholics on Disclosure, Support, and Assessment of Trustworthy Adults	Sweden	Interviews, individually or focus groups	To describe adolescents of alcoholics’ (COA) perspectives on disclosure and support	*n* = 27 aged 12–19 years Girls 24 Boys 3	Two themes presented: (1) Designing a Story and Risk Assessment of Adults and (2) Support from Adults, Trust-Distrust. COA reported assessing the trustworthiness of adults before disclosing their home situation. Before disclosure they may have raised their level of consciousness, told a peer, told an adult stranger, or indirectly communicated with an adult
**13.** **Wangensteen & Westby (2021)** Breaking the Cycle: Young Peoples’ Stories of Protection and Support While Growing up with Parental Substance use Disorder	Norway	In-depth interviews	To explore the narratives of young people regarding the circumstances that protected and supported them as they grew up around parental SUD during their childhood	*n* = 5 aged 21–26 years Female 4 Male 1	Safe living conditions, significant relationships and respectful and caring conversations with professionals seemed to be protective and supportive factors. This may have contributed to improving their everyday life and mental health, thus helping interrupt the intergenerational transmission of SUD in their families

ACOAs = Adult Children of Alcoholics; COA = Children of Alcoholic; SUD = substance use disorder.

### Analysis

#### Reading the Included Studies

Three reviewers (S.R., B.B., and A.S.S.) were involved in the process of extracting the data. S.R. repeatedly read the abstracts, introductions, results, discussions and conclusions, taking notes of metaphors and searching for meaning regarding the substantive concern (Noblit & Hare, 1988). All of the included studies appeared to largely emphasize risks and negative impacts related to PSUP, despite titles and abstracts giving rise to expectations about a resource-oriented approach. However, in line with the review question, we scrutinized the included studies for metaphors, key terms and concepts, as used by the authors in communicating their results – those expressing the adolescents viewed from a resource rather than a risk-oriented perspective. All identified metaphors, both first-order interpretations of quotes from adolescents (e.g., “Just to get it all out of me somehow”) and second-order interpretations by the study authors (e.g., “Eventually came to understand”), were extracted. We then conducted a metaphorical reduction with the aim of distilling the essence of text elements with sufficient depth and significance to convey the findings from the included primary studies (Noblit & Hare, 1988).

#### Determining How the Studies are Related

We determined how the studies were related, in the sense of meaning within a resource-oriented perspective. This was achieved by juxtaposing the metaphors concerning the adolescents’ health promotion. The studies related to each other in terms of adolescent's age and the context of PSUP. We found some similar reciprocal metaphors; above all, the metaphors conveyed a wide range of aspects of the adolescents’ experiences. Some participants lived with their parents, and others lived away from home; thus, both present and retrospective experiences were shared.

#### Translating the Studies Into One Another

S.R., B.B., and A.S.S. were involved in an idiomatic and interpretive translation, comparing the metaphors from one study with those of others (Noblit & Hare, 1988). First, we organized the findings in a matrix, performing initial line-by-line coding, starting with the oldest study, as none of the 13 studies stood out as particularly comprehensive. The matrix layout illustrates how metaphors are organized in columns corresponding to each study, as shown in the examples of the translation process provided in the Supplementary material (File S4).

Second, we conducted an initial analysis considering the entire material, through which process some preliminary themes crystallized. Third, we further translated the studies into each other through systematic and repeated readings of all metaphors in the matrix, with an emphasis on related similarities and differences, while searching for meaning within a resource-oriented approach. Related findings were organized across the studies in the horizontal rows of the matrix as part of an ongoing process. Throughout this process, we checked the context of the metaphors within the studies to ensure credibility, while translations emerged concurrently. This iterative process involved identifying meaning between the primary studies and their findings and subsequent comparisons of similar results across studies, culminating in interpretive findings of a reciprocal translation.

Two examples of the final translations from the translation process are presented in the Supplementary material (File S4). To illustrate the ongoing adjustments, one example: “Significant reciprocal and respectful interactions” represents a new understanding translated from metaphors across seven studies (4; 5; 8; 9; 11; 12; 13). This translation emerged from “Experience of support” to “Trusted support” and finally to “ Significant reciprocal and respectful interactions”, as metaphors were interpreted and relocated within the matrix. This is exemplified by the metaphor “reciprocal exchanges with interested and caring others”, which adds nuances to this translation by emphasizing the significance of reciprocal exchanges. Another example of a translation is “Realistic expectations of their parents”. The metaphor “I can’t really change him, I have to learn to live with it …”, was initially related to the emerging translation “Accept through increased understanding and openness”. It was later translated into “Realistic expectations of their parents” because of the meaning was interpreted as reflecting the adolescents’ expectations rather than the reasoning behind them, which the earlier translation implied. A visualization of the 24 final translations is provided in the Supplementary material (File S5).

### Synthesis

#### Synthesizing the Translations

The analyses and synthesis were separate but non-linear phases. We interpreted the metaphors through translations of expressions of the adolescents’ (first-order constructs) and the original authors’ perspectives (second-order constructs) into third-order constructs (our interpretive salutogenic framework, values and experiences). The set of translations represents one level of the meta-ethnographic synthesis (Noblit & Hare, 1988). S.R., B.B., and A.S.S. were involved in conducting the synthesis: to make a whole into something more than the parts alone implied moving beyond the findings of the individual studies to a second level of synthesis (France et al., 2019). In this interpretive process, a line-of-argument synthesis extended beyond the translations placing any similarities and dissimilarities into a new interpretive context; thus, the overarching metaphor “Designing their own story emerged”. The synthesis from translations into sub-themes, themes, and the final overarching metaphor expresses a logical connection between the process of translation and the interpretive findings, i.e., synthesizing the translations “Realizing their situation is not normal”, “Understanding addiction as an illness” and “Realizing it is not their fault” into the sub-theme “Awakening”, and together with “Adapting to circumstances” and “Opening up on their own terms”, further synthesized into the theme “Controlling before opening up”. The complete synthesis is visualized in the Supplementary material (File S5).

Throughout the process, the location of metaphors, translations, sub-themes and themes, as well as our understanding of their relationships, were adjusted several times illustrating how meta-ethnography works as a creative, non-linear and iterative method. For example, “trust” initially stood out as significant when synthesizing the meaning of translations into sub-themes, leading first to the sub-theme “trusted support”. This was later expanded into the theme, “Choosing trusted support”, which conveys more than its individual parts, synthesizing the sub themes “Finding a sense of connection”, “Adopting a different perspective” and “Being handed a pen”. Similarly, the concept of agency gradually emerged as an essential element reflecting adolescents’ ability to design their own story – corresponding to “what was hidden become apparent” (Noblit & Hare, 1988). The synthesis provided a new and expanded understanding of adolescents’ experiences of health promotion and well-being when living with PSUP.

#### Expressing the Synthesis

To express our line-by-argument synthesis, we started by presenting the overarching metaphor and its accounts. Then we presented the three main themes and their sub-themes in a storyline as they emerged through the synthesis. To present the results to academics, professionals and policymakers, emphasizing prevention and protection in the context of substance use, we deliberately shaped our vocabulary and concepts to emphasize a resources-oriented perspective comprehending adolescents’ expressions of health promotion.

## Results

Based on our findings from the 13 included studies expressing the adolescent study participants in a resource-oriented perspective, an overarching metaphor emerged: “Designing their own story”*.* The translation and synthesis process of findings from the included primary studies led to the identification and confirmation of this being overall core message capturing. The synthetic metaphor was interpreted as mediating a view on adolescents living with PSUP as competent agents navigating their lives and, over time, creating a narrative of their own health promotion in a complex and challenging situation. “Designing a story” involved many aspects of the adolescents’ lives, and they could draw on their inherent resources and those they perceived as accessible to move in a health-promoting direction. The metaphor “Designing their own story” is accentuated by three main themes: (1) “controlling before opening up”; (2) *“*choosing trusted support”; and (3) “learning for life”.

### Controlling Before Opening Up

“Controlling before opening up” is a phrase that reflects the studies’ messages of the adolescents’ needing and desiring to maintain control over their lives before disclosure, including their decisions of when, how, and to whom they disclose their experiences with PSUP. Disclosure of their home situation was often a gradual process accentuated in three sub-themes: (1) “adapting to circumstances”; (2) *“*awakening”; and (3) “opening up on their own terms”*.*

**“Adapting to circumstances”** reflects the adolescents’ conscious and unconscious adaptations to the unpredictable environment shaped by PSUP, demonstrating their capacity to activate resources of different kinds. The findings indicate strategies that contributed to a certain sense of control (2; 4; 10; 12), as expressed for example in this quote : “Somehow you've learned how to deal with it” (12). Such strategies could involve keeping the home situation a secret, especially when talking to others may involve exposing the adolescents’ own feelings. The adolescents expected both themselves and others to feel uncomfortable (2; 3; 4; 11) and sought to preserve their dignity by appearing “normal” in social settings (3). The latter is expressed in the following quote: “I prefer keeping things to myself” (11), thereby implying hiding feelings concerning home circumstances. The adolescents’ adaptation to control their parents was presented as a dynamic process changing over time; for example, during their adolescence, they could go from staying out of their parents’ way to confronting both parents with and those without substance use problems (1; 3; 10).

**“Awakening”** is a concept that reflects how the adolescents, in different ways and at different times, realized that they were part of an PSUP context and how PSUP affected themselves and the relationship with their parents. Their decision to keep secrets could be an indication that they already had realized that their situation was different. The insight could involve the realization of a home situation differing from other families due to their, of parents’ substance-use related behaviors (2; 4; 5; 10) and of PSUP not being their fault nor something they could influence (2; 4; 11; 12). The findings indicate that adolescents found it easier to cope when they viewed addiction as an illness (2; 10), which was seen to foster greater understanding and reduce blame toward their parents (5; 10; 11). When parents defined the premises of the adolescents’ reality, a shift was required to enable a new understanding of the truth. Altogether, it seemed like an awakening could appear as a catalyst for a turning point in the adolescents’ story, as expressed in this quote: “It made me realize that … you know … this situation is not going to define what I am going to be” (7).

**“Opening up on their own terms”** is a description of how adolescents carefully considered how, when and to whom they would disclose their situation during both the initial and subsequent disclosures. Disclosure sometimes became inevitable as the adolescents reported reaching the limit of what they could endure (3). Before opening up, the adolescents had expectations and assessed the trustworthiness and attitudes of adults, as well as to what extent the latter genuinely cared about them (3; 9; 11; 12; 13), as expressed in these quotes: “They asked questions about me, not about my parents” (13) or “Nobody asked, so I didn't care to tell” (9). Questions had to be posed cautiously (12; 13) and the adolescents could still remain concerned with maintaining privacy, being sensitive about what they shared (4; 12). To explore what it might be like to disclose their situation, adolescents sometimes turned to friends, strangers or even imaginary figures.

### Choosing Trusted Support

“Choosing trusted support” reflects the adolescents’ capacity to recognize empowering and supportive interactions and identify whom they could trust. They selected both imaginary and real, present and absent significant others as resources for various roles. Support could last for varying durations and provided different levels of assistance, including advice, care, and a sense of security. This theme is accentuated by three sub-themes: (1) “finding a sense of connection”; (2) “adopting different perspectives”; and (3) “being handed a pen”.

“**Finding a sense of connection**” indicate that the adolescents, without necessarily disclosing their situation, needed someone to be present and supportive in daily life. A sense of belonging and safety in relationships fostered a feeling of connectedness (7; 8; 9) and these valued interactions were marked by reciprocity and trust (5; 6; 7; 8; 12; 13) without necessarily including adolescents’ parents. Furthermore, developing a shared understanding (2; 4; 7; 11; 12; 13), being cared for (7; 8; 13), having fun together (5; 11) and telling the truth (7; 12) emerged as key elements contributing to a sense of connectedness. This sense could influence the adolescents’ receptiveness to talking, sharing, receiving and engaging with the support offered. To quote a typical expression, “To know that they're not getting anything out of it, but they're still going to be there for you” (9). Family members and relatives, mothers, fathers, non-using parents, siblings, teachers, neighbors, friends, professionals, pastors and parental figures could undertake the role of support on their own initiative (1; 3; 4; 5; 7; 9; 12; 13). Additionally, the adolescents actively and successfully reached out to peers, adults, siblings, family members, professionals and imaginary others for support (4; 5; 6; 7; 9; 10; 12), while some seemed to drive themselves forward without any support (10). Interestingly, non-human systems could play a significant role in this regard with imaginary others and inanimate objects fostering completely internal dialogues (Mushonga & van Breda, 2021).

**“Adopting different perspectives”** is a strategy that reflects the circumstance that interactions with imaginary or real, present or non-present others were perceived as potential for opening for new insights and learning. Some adolescents experienced that health services had changed the way they thought and felt by helping them learn about addiction and their own emotions (11; 13). Many benefited from sharing experiences with others in the same situation, as expressed in this quote: “see it from their point of view” (11). Listening to motivational speakers and reading could help them recognize their own worthiness and reduce feelings of guilt and self-defeat concerning a sense of being responsible for their parents’ substance use problems (6; 7; 10), as expressed in this quote: “It was a really good experience to meet other people, to see it from their point of view and to talk openly about what's happening without fear of being judged” (10). This also applied to the value of positive role models, such as non-using parents (1; 9). Messages from songwriters, authors and speakers could also guide the adolescents, identifying both obstacles and opportunities (6; 7).

**“Being handed a pen”** is a metaphor that communicates the importance of adolescents experiencing that someone had faith in them and empowered them through expectations even in their stressful situation. Actions initiated by others, such as providing material support, offering emotional support and presenting challenges, could serve as catalysts for resolve, hope, and encouragement (6; 7). The adolescents needed someone to acknowledge their internal resources and capacity for coping, motivating them to change, providing regardless of their circumstances, as highlighted by this statement: “There is someone who tells you that even in that situation, you still have to have goals” (9). Their internal imaginary conversations with fictional friends and non-present figures through music, written texts and inspirational speakers motivated them to persevere. Engagement with symbolic objects symbolizing peace or reminding them of happy moments was significant in this regard (6).

### Learning for Life

The theme “learning for life” reflects how the adolescents experienced both positive and negative life events, developing these into meaningful life experiences that shaped their understanding, values and choices leading them in a health-promoting direction. New stories were further shaped by new understandings and courage, reflecting a process of awakening and serving as a natural step in the transition to adulthood. This theme is accentuated by two sub-themes: (1) “moving forward through acceptance” and (2) “sense of agency”.

**“Moving forward through acceptance”** reflects the adolescents’ life experiences of separating from their parents. The findings indicate that the adolescents re-evaluated their own and their parents’ roles, behaviors and attitudes both during adolescence and in retrospect as young adults (1; 2; 3; 7; 10; 11). This process involved acceptance, self-compassion, changes in values, and a reframing of previous experiences and cherished moments as strengths that increased well-being and facilitated letting go of painful past experiences while opening up new perspectives and opportunities.

Many of the adolescents had a loving but complicated relationship with their parents (1; 7; 13), as expressed in this quote: “… it still felt like my mother was very loving towards us even when she was drunk” (1). Increased understanding and openness about PSUP made it easier for the adolescents to have realistic expectations (2; 3; 5; 10; 11), as expressed in this quote: “I'm just like, yeah, this is my momma” (5). In this process, the adolescents came to realize that their parents were not their responsibility (2; 11), as expressed in this quote: “…can't really change him, I have to learn to live with it.” (11). Through this process, the adolescents could also realize their own worth and needs (2; 3; 6; 10; 11), developing a deeper understanding of their own emotions, along with an increased ability to regulate emotions (6; 11). Acceptance also helped them make different choices and life decisions than their parents based on their experiences living with PSUP (2; 9; 10).

**“Sense of Agency”** The studies showed that along with challenges, the adolescents could experience well-being, developing confidence and comfort in moving forward (2; 6; 10; 11; 13), as expressed in these quotes: “I'm well-equipped to face adversity later in life” and “I really break the cycle of problems in my family” (13). The adolescents experiencing a sense of agency could develop strategies for managing their life situation, such as practicing the role of a young carer and motivating themselves, acting strategically, making conscious decisions, and taking control over what they did and whom they involved. They could assess their own situation and participate in decision-making (1; 3; 6; 8; 11; 12). They also tried to take responsibility for themselves and their siblings’ safety and well-being (3; 7; 8). Their sense of agency became visible when they searched for attention, understanding and comfort from others and became aware of skills and approaches that empowered them to navigate challenges (9; 11).

The findings also indicate that adolescents’ agency was expressed through increased self-awareness expressing themselves through their narrative voice (5; 11) and sharing their stories, as expressed in this quote: “Just to get it all out of me somehow” (12). Expressing themselves through creative activities and engaging in conversations with trusted individuals, both externally and internally, further enhanced their sense of agency as well. Some adolescents further influenced their own situation by making efforts to view their lives in a positive light; for example, by trying to: “praise myself when I do well, such as ‘you did a good job’” (10).

Though the adolescents’ life situation and experiences varied, our findings indicated that adolescents with current PSUP experience expressed more strongly their need for control and privacy, taking responsibility and developing their own way of coping. In retrospect, adolescents expressed more acceptance, a greater feeling of control and less blame, and acknowledged that their life experiences had given them skills and confidence to handle life. However, the adolescents engaging in support groups expressed a process of learning, more the same as adolescents living with PSUP in retrospect, even if they still lived with PSUP.

## Discussion

The aim of this meta-ethnography was to expand the existing knowledge through synthesizing qualitative studies on adolescents’ experiences living with PSUP from a salutogenic health-promoting perspective, focusing on resources for positive health. The overarching metaphor “Designing their own story” symbolizes that the adolescents were competent agents navigating and creating their own narrative in the context of PSUP. In the following sections, we discuss, through a salutogenic framework, how the adolescents were able to progress even in such a challenging life situation. Furthermore, we discuss how these findings add new insights and perspectives to complement previous studies, which have often focused primarily on the adversities faced by adolescents in such situations. Based on our findings, we propose ways to offer adolescents tailored support through a whole-person perspective that emphasizes capacity building, activates personal resources and acknowledges adolescents as competent agents.

### How was it Even Possible for Adolescents with PSUP to Move Forward as Competent Agents in Their Life Situation?

From a salutogenic health-promoting perspective, one might expect adolescents living with PSUP to move toward breakdown on the “disease” end of the health continuum, due to feeling unsafe and facing a highly demanding, chronic life situation (Antonovsky, 1987). Previous studies show that children and adolescents in such a context feel insecure and afraid of being alone, struggle with emotions and close relationships (Bönnhoff & Larsen, 2014; Haverfield & Theiss, 2014; Järvinen, 2015; Werner & Malterud, 2016) and are left with no choice but to take care of themselves (Reupert et al., 2012; Ronel & Haimoff- Ayali, 2010; Ronel & Levy-Cahana, 2011; Templeton et al., 2009). Our findings, focusing on resources, nonetheless show that the adolescents were also able to feel confident and comfortable caring for themselves, managing challenges, and experiencing well-being.

“Controlling before opening up” may then be understood as an expression of strength, resonating with findings on children as next of kin conceptualizing themselves as relational agents through their embodied practices of being and acting (Ytterhus et al., 2024) and young carers in which responsibility may empower but also harm if excessive (Kallander et al., 2018; Leu & Becker, 2019). Alongside research on controlling the uncontrollable (Muir et al., 2023) and agency over one's destiny (Orford et al., 2010), our findings hightlights agency as a strength and which actions adolescents can be enabled to do in order to take part within health-promoting contexts. This may represent an approach that acknowledges stress as potentially pathogenic, neutral or health-promoting, depending on the availability and use of resources, as well as the individual's process of meaning-making, comprising a perspective that aligns with human agency within health promotion emphasizing active engagement with the environment in pursuit of one's goals (Cross & Woodall, 2024).

Consistent with previous research emphasizing the importance of social support (Ashton et al., 2021; Cross & Woodall, 2024; Haugland et al., 2025; Ytterhus et al., 2024), our findings also underscore its significance as a resource related to health and well-being of adolescents dealing with PSUP. Novelly, some adolescents chose imaginary or absent significant others (e.g., dolls) as meaningful sources supporting their sense of agency. This finding may offer an expended perspective compared to other research that refers to dialogues with pets or toys in the context of isolation and loneliness among adolescents living with PSUP (Muir et al., 2023), indicating non-human systems can act as health-promoting resources.

Focusing on the adolescents’ narratives, we found they transitioned from adapting to their parents’ substance use and reality to questioning and redefining their story and their identity. This finding may relate to the coping strategy “withdraw from it” of Orford et al. (2010), referring to actions involving increased independence and distance, avoiding self-sacrifice and resignation. While the strategies of Orford et al. (2010) often reflect dilemmas about how to respond to drinking or drug behaviors, our findings suggest a longer-term process in which experiences of adaptation could be a resource to achieve goals later in life.

Many adolescents developed self-efficacy through life experiences, along with the expectation of being able to handle life. This finding is consistent with salutogenesis normalizing stressors and situations of adversity. In the salutogenic metaphor “river of life”, everyone is constantly interacting with the environment and encounters waves, whirlpools and calm waters (Antonovsky, 1987). From this point of view, adolescents living with PSUP can, similar to anyone else, learn to navigate life and strengthen their identities through identifying and utilizing available resources. In the context of PSUP, it is worth questioning whether coping strategies are always health-promoting, as they may involve managing risk or avoidance of high-stress situations (Ahlborg et al., 2024; Backett-Milburn et al., 2008), even putting adolescents in danger (Arai et al., 2021), or contributing to negative mental health outcomes, antisocial behavior or self-harm (Muir et al., 2023). These findings may clarify the role of recognizing health-promoting resources, while still underscore the importance of understanding and prevent risks and distress.

### How Can Adolescent’s Resources be Activated to Enable Them to Tell Their Story in a Health-promoting Direction?

We found that adolescents living with PSUP could engage in an ongoing project of maintaining a sense of control amid chaos in everyday life: such engagement which could emerge into strength in other and future contexts. The adolescents could be both competent and willing agents designing their own story. These findings highlight the particular significance of the attitudes of those involved in the adolescents’ lives, underscoring key health promotion values such as empowerment, autonomy, involvement and participation (Cross & Woodall, 2024).

Notably, the adolescents valued expectations and responsibilities, highlighting the importance of showing trust in their potential regardless of their home situation. These findings may extend current knowledge about children and young people in similar contexts who encounter stigma and prejudice from professionals (Muir et al., 2023), feel insufficiently cared for, have limited trust in adults and perceive available support as unhelpful (Backett-Milburn et al., 2008; Ronel & Haimoff-Ayali, 2010; Templeton et al., 2009). Viewing adolescents living with PSUP from a salutogenic, health-promoting perspective involves acknowledging them as adolescents trying to navigate stressful and chaotic environments, much like their peers. This perspective may enable professionals, service providers and society to understand these adolescents beyond their PSUP.

The findings demonstrate that adolescents living with PSUP draw on a wide range of resources. Langeland and Vinje (2022) highlight the importance of acknowledging and identifying resources together with individuals as a guiding principle for salutogenic health-promoting interventions. According to the whole-person approach emphasizing the unique life story, professionals can facilitate awakening, disclosure and autonomy by supporting adolescents living with PSUP in recognizing their coping narrative, potential and identity. This involves exploring questions such as “Who are you?” and “What is important to you?”, comprising questions that extend beyond mapping symptoms and adverse experiences. In this perspective, health and well-being are more than just the absence of, or low levels of, risk factors: they also include imagination, love, play, meaning and will (Antonovsky, 1996).

Another finding is that encouraging adolescents to tell their story in a health-promoting direction involves a gradual learning process, with support needs varying across stages. Professionals must be sensitive to context and offer tailored, responsive support while navigating the dilemma of enabling adolescents to stay in control yet accept the support provided.

Overall, this meta-ethnography underscores that a different story emerges when the focus is explicitly on salutary health promotion. Different perspectives on health promotion and coping may indicate what professionals emphasize when interacting with adolescents living with PSUP and their stories. The limited findings concerning health-promoting factors may reflect the traditional focus on prevention, protection and the impact of risk factors related to PSUP. However, pathogenic and salutogenic perspectives as complementary (Antonovsky, 1987) and both perspectives can elicit stories that deserve to be acknowledged and supported.

While there are studies addressing aspects of health promotion, no study has to our knowledge been conducted with the primary aim of clarifying health promotion related to PSUP. Further research is needed to better understand how to support these adolescents from their own perspective in a health-promoting direction. Such research must recognize that, regardless of where adolescents living with PSUP are on the health continuum, there will be potential to improve their health and well-being within their stressful and challenging life situation.

### Strengths and Limitations

The strengths of this meta-ethnography lie in its rigorous methodology, comprehensive literature review, as well as its capacity to generate new insights (France et al., 2019; Noblit and Hare, 1988). The development of translations, sub-themes, themes and the overarching metaphor was guided by iterative discussions during team meetings. The use of an overarching metaphor to symbolize adolescents’ expressions may offer a richer and more nuanced understanding of the concerned adolescents’ experiences in everyday life. Compared with prior research on adolescents living with PSUP, the findings in this meta-ethnography highlight an additional narrative. They clarify how perspectives on health matter for understanding different aspects of reality and the opportunities they offer to expand our understanding of the adolescents’ situations and needs.

A limitation of this study may be the scarce empirical data concerning health promotion in the included primary studies. Furthermore, the articles were predominantly understood as interpreted from a risk prevention perspective. However, a strength of this study is that it explores an underexplored perspective focusing on health promotion among adolescents living with PSUP, even amid stories of adverse experiences.

Studies focusing exclusively on PSUP were deliberately selected, as this life situation is complex and characterized by specific features such as unpredictability, disruption, economic challenges, and significant stigma and shame. The inclusion of studies involving comorbidity with other disorders or illnesses might have overshadowed these aspects. A potential limitation of this approach is that nuances of PSUP may have been lost, given that substance use and mental health are often considered as closely interconnected in the professional context.

## Conclusions

This meta-ethnography was aimed to expand the existing knowledge through synthesizing qualitative studies on adolescents’ experiences living with PSUP from a salutogenic health-promoting perspective, focusing on resources for positive health.

The overarching metaphor “Designing their own story” symbolizes how adolescents living with PSUP demonstrated agency in a stressful and challenging life situation. They could be in a continuous process where life experiences shaped their identity and increased their autonomy. Furthermore, adolescents living with PSUP could be both competent and willing to take steps on their own, indicating a sense of control over their actions and actively choosing whom to involve for support.

Overall, this meta-ethnography provides useful insights on the potential of adolescents living with PSUP to navigate their everyday lives and to design their own story based on all their experiences. This potential may indicate the importance of maintaining positive expectations regarding adolescents’ agency and collaboratively exploring their resources. Simultaneously, it is essential that professionals and services offer interventions with opportunities for the adolescents to shape, tell and support their own narrative by acknowledging the responsibility of the municipalities to recognize both the need for and the benefits of tailored support in facilitating health promotion.

## Supplemental Material

sj-pdf-1-nad-10.1177_14550725261436976 - Supplemental material for Designing their Own Story: A Meta-Ethnography of Health Promotion Among Adolescents with Parental Substance Use Problems Supplemental material, sj-pdf-1-nad-10.1177_14550725261436976 for Designing their Own Story: A Meta-Ethnography of Health Promotion Among Adolescents with Parental Substance Use Problems by Signegun Romedal, Anne Schanche Selbekk, Siri Håvås Haugland, John-Kåre Vederhuus and Bente Birkeland in Nordic Studies on Alcohol and Drugs

sj-pdf-2-nad-10.1177_14550725261436976 - Supplemental material for Designing their Own Story: A Meta-Ethnography of Health Promotion Among Adolescents with Parental Substance Use Problems Supplemental material, sj-pdf-2-nad-10.1177_14550725261436976 for Designing their Own Story: A Meta-Ethnography of Health Promotion Among Adolescents with Parental Substance Use Problems by Signegun Romedal, Anne Schanche Selbekk, Siri Håvås Haugland, John-Kåre Vederhuus and Bente Birkeland in Nordic Studies on Alcohol and Drugs

sj-pdf-3-nad-10.1177_14550725261436976 - Supplemental material for Designing their Own Story: A Meta-Ethnography of Health Promotion Among Adolescents with Parental Substance Use Problems Supplemental material, sj-pdf-3-nad-10.1177_14550725261436976 for Designing their Own Story: A Meta-Ethnography of Health Promotion Among Adolescents with Parental Substance Use Problems by Signegun Romedal, Anne Schanche Selbekk, Siri Håvås Haugland, John-Kåre Vederhuus and Bente Birkeland in Nordic Studies on Alcohol and Drugs

sj-pdf-4-nad-10.1177_14550725261436976 - Supplemental material for Designing their Own Story: A Meta-Ethnography of Health Promotion Among Adolescents with Parental Substance Use Problems Supplemental material, sj-pdf-4-nad-10.1177_14550725261436976 for Designing their Own Story: A Meta-Ethnography of Health Promotion Among Adolescents with Parental Substance Use Problems by Signegun Romedal, Anne Schanche Selbekk, Siri Håvås Haugland, John-Kåre Vederhuus and Bente Birkeland in Nordic Studies on Alcohol and Drugs

sj-pdf-5-nad-10.1177_14550725261436976 - Supplemental material for Designing their Own Story: A Meta-Ethnography of Health Promotion Among Adolescents with Parental Substance Use Problems Supplemental material, sj-pdf-5-nad-10.1177_14550725261436976 for Designing their Own Story: A Meta-Ethnography of Health Promotion Among Adolescents with Parental Substance Use Problems by Signegun Romedal, Anne Schanche Selbekk, Siri Håvås Haugland, John-Kåre Vederhuus and Bente Birkeland in Nordic Studies on Alcohol and Drugs
