# Supplementary material for: Designing their Own Story: A Meta-Ethnography of Health Promotion Among Adolescents with Parental Substance Use Problems
Source: Nordisk Alkohol Nark. 2026 Apr 17;43(3):235–60. doi: 10.1177/14550725261436976 (PMC13090238; doi:10.1177/14550725261436976)

## Supplementary File 1

**Illustration of seven non-linear and iterative phases in Meta-ethnography**

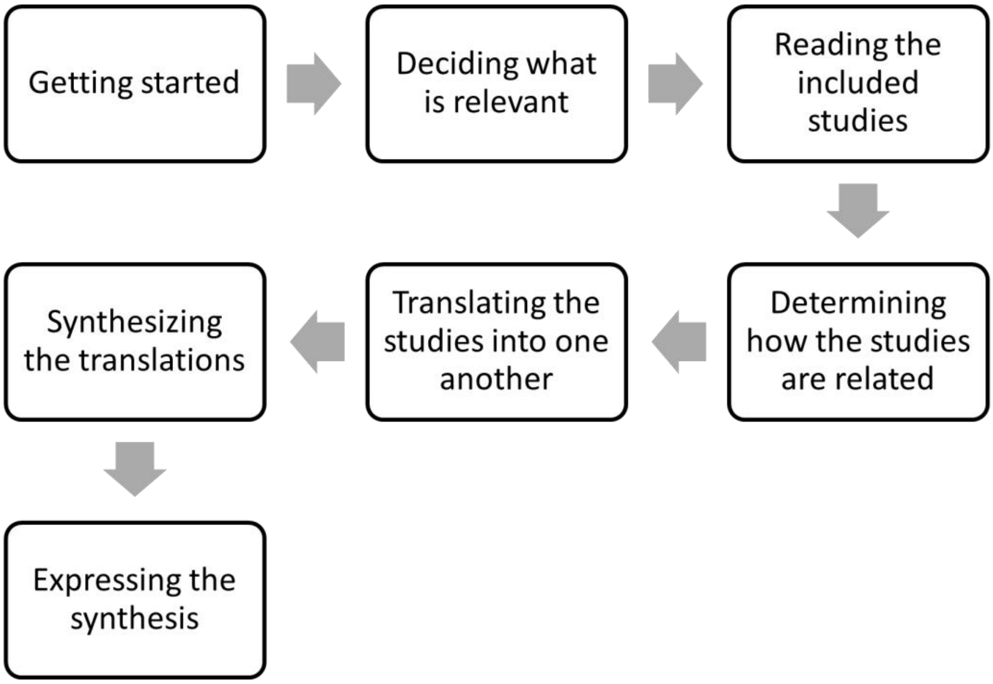

Supplement: sj-pdf-1-nad-10.1177_14550725261436976 - Supplemental material for Designing their Own Story: A Meta-Ethnography of Health Promotion Among Adolescents with Parental Substance Use Problems [file sj-pdf-1-nad-10.1177_14550725261436976.pdf]
