# Supplementary material for: Designing their Own Story: A Meta-Ethnography of Health Promotion Among Adolescents with Parental Substance Use Problems
Source: Nordisk Alkohol Nark. 2026 Apr 17;43(3):235–60. doi: 10.1177/14550725261436976 (PMC13090238; doi:10.1177/14550725261436976)
Supplement: sj-pdf-2-nad-10.1177_14550725261436976 - Supplemental material for Designing their Own Story: A Meta-Ethnography of Health Promotion Among Adolescents with Parental Substance Use Problems [file sj-pdf-2-nad-10.1177_14550725261436976.pdf]

Supplementary File 1

Search strategy

Embase

1 "children of alcoholics"/ (172)

2 ((child* or offspring or adolescent or teenager or youth or young* or son or sons or daughter*) adj2 alcoholi*).tw,kw. (1814)

3 ((parent* or maternal or paternal or mother* or father*) adj3 drinking).tw,kw. (1898)

4 ((child* or offspring or adolescent or teenager or youth or young* or son or sons or daughter*) adj3 (parent* or maternal or paternal or mother* or father*) adj3 ("use" or "using" or abus* or misus* or dependen* or addict*) adj3 (substance* or drug* or sedative* or opiate* or opioid*)).tw,kw. (733)

5 1 or 2 or 3 or 4 (4356)

6 limit 5 to ("qualitative (maximizes sensitivity)" and english and (article or article in press)) (492)

Cinahl

S1 (MH "Children of Alcoholics") Search modes - Find all my search terms 236

S2 TI ((((child* or offspring or adolescent or teenager or youth or young* or son or sons or daughter*) W2 alcoholi*))) OR AB ((((child* or offspring or adolescent or teenager or youth or young* or son or sons or daughter*) W2 alcoholi*))) Search modes - Find all my search terms 525

S3 TI ((((parent* or maternal or paternal or mother* or father*) N3 drinking))) OR AB ((((parent* or maternal or paternal or mother* or father*) N3 drinking))) Search modes - Find all my search terms 875

S4 TI ((((child* or offspring or adolescent or teenager or youth or young* or son or sons or daughter*) N3 (parent* or maternal or paternal or mother* or father*) N3 ("use" or "using" or abus* or misus* or dependen* or addict*) N3 (substance* or drug* or sedative* or opiate* or opioid*)))) OR AB ((((child* or offspring or adolescent or teenager or youth or young* or son or sons or daughter*) N3 (parent* or maternal or paternal or mother* or father*) N3 ("use" or "using" or abus* or misus* or dependen* or addict*) N3 (substance* or drug* or sedative* or opiate* or opioid*)))) Search modes - Find all my search terms 795

S5 S1 OR S2 OR S3 OR S4Limiters - Peer Reviewed; Language: English; Clinical Queries: Qualitative - Best Balance

Search modes - Find all my search terms 178

PsycInfo

1 Children of alcoholics/ (948)
2 ((child* or offspring or adolescent or teenager or youth or young* or son or sons or daughter*) adj2 alcoholi*).tw,id. (2275)
3 ((parent* or maternal or paternal or mother* or father*) adj3 drinking).tw,id. (1268)
4 ((child* or offspring or adolescent or teenager or youth or young* or son or sons or daughter*) adj3 (parent* or maternal or paternal or mother* or father*) adj3 ("use" or "using" or abus* or misus* or dependen* or addict*) adj3 (substance* or drug* or sedative* or opiate* or opioid*)).tw,id. (958)
5 1 or 2 or 3 or 4 (4465)
6 limit 5 to ("qualitative (maximizes sensitivity)" and "0110 peer-reviewed journal" and english) (829)

Academic Search

S1 TI (((child* or offspring or adolescent or teenager or youth or young* or son or sons or daughter*) W2 alcoholi*)) OR AB (((child* or offspring or adolescent or teenager or youth or young* or son or sons or daughter*) W2 alcoholi*)) Search modes - Find all my search terms 1,155

S2 TI (((parent* or maternal or paternal or mother* or father*) N3 drinking)) OR AB (((parent* or maternal or paternal or mother* or father*) N3 drinking)) Search modes - Find all my search terms 1,457

S3 TI (((child* or offspring or adolescent or teenager or youth or young* or son or sons or daughter*) N3 (parent* or maternal or paternal or mother* or father*) N3 ("use" or "using" or abus* or misus* or dependen* or addict*) N3 (substance* or drug* or sedative* or opiate* or opioid*))) OR AB (((child* or offspring or adolescent or teenager or youth or young* or son or sons or daughter*) N3 (parent* or maternal or paternal or mother* or father*) N3 ("use" or "using" or abus* or misus* or dependen* or addict*) N3 (substance* or drug* or sedative* or opiate* or opioid*))) Search modes - Find all my search terms 1,385

S4 S1 OR S2 OR S3 Limiters - Peer Reviewed; Language: English

Search modes - Find all my search terms 3,342

S5 TI ((interview* or qualit*)) OR AB ((interview* or qualit*)) Search modes - Find all my search terms 2,353,840

S6 S4 AND S5 Search modes - Find all my search terms 584

Web of Science

((child* or offspring or adolescent or teenager or youth or young* or son or sons or daughter*) NEAR/2 alcoholi*) (Topic) or ((parent* or maternal or paternal or mother* or father*) NEAR/3 drinking) (Topic) or ((child* or offspring or adolescent or teenager or youth or young* or son or sons or daughter*) NEAR/3 (parent* or maternal or paternal or mother* or father*) NEAR/3 ("use" or "using" or abus* or misus* or dependen* or addict*) NEAR/3 (substance* or drug* or sedative* or opiate* or opioid*)) (Topic) and interview OR qualitat* (Topic) and Article (Document Types) and English (Languages) (3627)
