# Supplementary material for: Designing their Own Story: A Meta-Ethnography of Health Promotion Among Adolescents with Parental Substance Use Problems
Source: Nordisk Alkohol Nark. 2026 Apr 17;43(3):235–60. doi: 10.1177/14550725261436976 (PMC13090238; doi:10.1177/14550725261436976)
Supplement: sj-pdf-3-nad-10.1177_14550725261436976 - Supplemental material for Designing their Own Story: A Meta-Ethnography of Health Promotion Among Adolescents with Parental Substance Use Problems [file sj-pdf-3-nad-10.1177_14550725261436976.pdf]

Supplementary File 3

Critical appraisal for included studies (CASP)

Critical appraisal questions										
Article	1	2	3	4	5	6	7	8	9	10
Bickelhaupt et al. (2021)	Y	Y	Y	C	Y	C	N	Y	Y	Y
Holmila et al. (2011)	C	Y	C	Y	Y	N	Y	Y	Y	C
O'Connor et al. (2014)	Y	Y	Y	Y	Y	N	Y	Y	Y	Y
Alexanderson & Näsman (2017)	Y	Y	Y	Y	Y	N	C	Y	Y	Y
Park & Schepp (2017)	Y	Y	Y	Y	Y	N	Y	Y	Y	Y
Hagström & Forinder (2022)	Y	Y	Y	Y	Y	C	Y	Y	Y	Y
Tinnfält et al. (2011)	Y	Y	Y	Y	Y	Y	Y	Y	Y	Y
Offiong et al. (2020)	Y	Y	Y	C	Y	Y	C	Y	Y	Y
Templeton et al. (2011)	Y	Y	Y	C	Y	N	Y	Y	Y	Y
Mushonga & van Breda (2023)	Y	Y	Y	Y	Y	N	Y	C	Y	C
Mushonga & van Breda (2021)	Y	Y	Y	C	Y	N	Y	C	Y	C
Johnson (2013)	Y	Y	Y	C	Y	N	N	Y	Y	Y
Wangensteen & Westby (2021)	Y	Y	Y	Y	Y	C	Y	C	Y	C

Critical appraisal questions: (1) Was there a clear statement of the aim of the research? (2) Is a qualitative methodology appropriate? (3) Was the research design appropriate to address the aims of the research? (4) Was the recruitment strategy appropriate to the aims of the research? (5) Was the data collected in a way that addressed the research issue? (6) Has the relationship between researcher and participants been adequately considered? (7) Have ethical issues been taken into consideration? (8) Was the data analysis sufficiently rigorous? (9) Is there a clear statement of findings? (10) How valuable is the research? Y = yes, N = no, C = can't tell.
