# Supplementary material for: Designing their Own Story: A Meta-Ethnography of Health Promotion Among Adolescents with Parental Substance Use Problems
Source: Nordisk Alkohol Nark. 2026 Apr 17;43(3):235–60. doi: 10.1177/14550725261436976 (PMC13090238; doi:10.1177/14550725261436976)
Supplement: sj-pdf-4-nad-10.1177_14550725261436976 - Supplemental material for Designing their Own Story: A Meta-Ethnography of Health Promotion Among Adolescents with Parental Substance Use Problems [file sj-pdf-4-nad-10.1177_14550725261436976.pdf]

Supplementary File 4

The translation process (examples)

Tinnfält et al. (2011) Index paper	Templeton et al. (2011)	Holmila et al. (2011)	Johnson (2013)	O'Connor et al. (2014)	Alexanderson & Näsman (2017)	Park & Schepp (2017)	Offiong et al. (2020)	Bickelhaupt et al. (2021)	Mushonga & van Breda (2021)	Wangensteen & Westby (2021)	Hagström & Forinder (2022)	Mushonga & van Breda (2023)	**Translation**
act in a trust-worthy way	unobtrusive support provided in a non-stigmatizing way	under-standing from adults or peers, who don't spread confi-dential informa-tion to outsiders or to parents	positive interaction s in the form of open communi-cation	-	-	-	the sense of connect-edness	-	comfort each other and advise each other to move on with their lives	respectful and caring conversations with professionals	-	reciprocal exchanges with interested and caring others	Significant reciprocal and respectful interactions
	(1) " I can't really change him, I have to learn to live with it.."<br><br>(2) realistic expectations of their parents…, not trying to change their parents' behavior	-	"I'm just like, yeah, this is my momma"	-	-	gradually open their minds to think of their fathers positively and approach them as they are	-	gaining more feelings of control in the relationship with their alcoholic parent	-	their relationship with their parents were discussed as loving, but complicated	better under-standing of parental substance use	-	Realistic expectations of their parents
