# Supplementary material for: Designing their Own Story: A Meta-Ethnography of Health Promotion Among Adolescents with Parental Substance Use Problems
Source: Nordisk Alkohol Nark. 2026 Apr 17;43(3):235–60. doi: 10.1177/14550725261436976 (PMC13090238; doi:10.1177/14550725261436976)
Supplement: sj-pdf-5-nad-10.1177_14550725261436976 - Supplemental material for Designing their Own Story: A Meta-Ethnography of Health Promotion Among Adolescents with Parental Substance Use Problems [file sj-pdf-5-nad-10.1177_14550725261436976.pdf]

Supplementary File 5

Translations into sub-themes, themes, and overarching metaphor

Translations	Sub-themes	Themes	Overarching metaphor
Strategies for coping with everyday life<br>Wants to be like everyone else<br>Changing strategies during time	Adapting to circumstances		
Realizing their situation is not normal<br>Understanding addiction as an illness<br>Realizing it is not their fault	Awakening	Controlling before opening up	
Assessing trustworthiness and attitudes<br>Conscious choices<br>Expectations and demands about how adults should behave and proceed	Opening up on their own terms		
Significant reciprocal and respectful interactions<br>Someone being there for them over time<br>Feelings of belonging trough shared understanding	Finding a sense of connection		
Support from the services<br>Sharing experiences with others in a similar situation<br>Role models and advice from people, present or absent, and from nonhuman systems	Adopting different perspectives	Choosing trusted support	**Designing their own story**
Guiding empowering messages<br>Faith through expectation<br>Support as trigger for resolve, hope and encouragement	Being handed a pen		
Realistic expectations of their parents<br>Accept through increased understanding and openness<br>Finding their own identity<br>Reviewing their experiences from the past	Moving forward through acceptance		
Expressing themselves in different ways<br>Able to take part in decision-making<br>Mobilizing energy to cope<br>Reaching out for help and support<br>Self-efficacy for future challenges	Sense of agency	Learning for life	
